# Clinical outcomes and perioperative safety of primary total knee arthroplasty for hemophilic arthropathy versus osteoarthritis: a retrospective cohort study

**DOI:** 10.1186/s42836-026-00431-5

**Published:** 2026-09-18

**Authors:** Ruixiang Ma, Quan Liu, Xianli Hu, Haibo Sun, Jing Zhao, Juan Tong, Zheng Su, Chen Zhu

**Affiliations:** 1 Department of Orthopedics, Centre for Leading Medicine and Advanced Technologies of IHM, The First Affiliated Hospital of USTC, Division of Life Sciences and Medicine, University of Science and Technology of China, Hefei, Anhui 230001 China; 2 Department of Rehabilitation, The First Affiliated Hospital of USTC, Division of Life Sciences and Medicine, University of Science and Technology of China, Hefei, Anhui 230001 China; 3 Department of Hematology, The First Affiliated Hospital of USTC, Division of Life Sciences and Medicine, University of Science and Technology of China, Hefei, Anhui 230001 China

**Keywords:** Hemophilic arthropathy, Osteoarthritis, Total knee arthroplasty, Coagulation factor replacement therapy

## Abstract

**Background:**

Hemophilic arthropathy (HAr) frequently progresses to end-stage joint disease requiring total knee arthroplasty (TKA), but coagulation dysfunction raises concerns about perioperative bleeding and complications. We aim to analyze the clinical outcomes and safety associated with primary TKA in patients with HAr.

**Methods:**

This single-center retrospective cohort included 22 TKAs in 20 men with HAr and 66 TKAs in 66 male osteoarthritis (OA) controls selected by surgeon and proximity of surgery date. The procedure was the analysis unit, and standard errors were clustered by patient. Continuous outcomes were compared with Welch tests and adjusted using baseline-outcome analysis of covariance including group, age, and body mass index. Sparse binary outcomes were exploratory and analyzed with Fisher exact tests and Firth logistic regression. Calculated blood loss was evaluated using the Nadler-Gross method.

**Results:**

Patients with HAr were younger (41.86 ± 11.29 versus 68.33 ± 6.70 years). Mean improvement in the Knee Society clinical score was 49.64 ± 18.43 versus 53.24 ± 5.77 points (unadjusted between-group change-score difference, − 3.61; 95% CI − 11.88 to 4.67; *p* = 0.376). Adjusted 12-month group effects were 0.54 points (95% CI − 4.19 to 5.28; *p* = 0.820) for Knee Society clinical score and 3.02 points (95% CI − 9.73 to 15.77; *p* = 0.639) for Knee Society function while adjusted flexion remained lower (mean difference, − 19.64°; 95% CI − 38.29° to − 0.99°; *p* = 0.039). Calculated blood loss did not differ significantly (532.53 ± 355.18 versus 628.00 ± 328.38 mL; *p* = 0.275), while transfusion occurred in 4/22 versus 0/66 procedures (*p* = 0.003). Wound problems (6/22 versus 3/66) and 90-day readmissions (9/22 versus 1/66) were more frequent in crude comparisons, but adjusted estimates were highly imprecise.

**Conclusions:**

Under comprehensive blood management, TKA produced substantial within-group functional improvement in HAr. Adjusted 12-month Knee Society clinical and function scores were not detectably different, although flexion remained lower. Calculated blood loss was not increased, but transfusion and unadjusted wound or readmission signals warrant multidisciplinary care and confirmation in larger, prospectively defined cohorts.

**Supplementary Information:**

The online version contains supplementary material available at https://doi.org/10.1186/s42836-026-00431-5.

## Background

Hemophilia is an X-linked recessive genetic bleeding disorder caused by deficiency of coagulation factor VIII or IX [1]. The most common bleeding sites in hemophilia are the joints and muscles, with approximately 80% of cases involving joint bleeding [2]. From 2007 to 2019, the National Hemophilia Case Information Management System reported 17,779 cases of Hemophilia A, with joint bleeding accounting for 61% [3]. Recurrent hemarthrosis drives synovial hypertrophy, cartilage loss, subchondral change, and progressive hemophilic arthropathy (HAr). When joint pain, severe deformity, and functional impairment significantly reduce the patients’ quality of life, surgical intervention becomes necessary. Advances in factor-replacement therapy and multidisciplinary perioperative management have made total knee arthroplasty (TKA) an increasingly feasible treatment for end-stage HAr. Yet TKA in this population still requires coordinated hematology, anesthesia, rehabilitation, and surgical care because of the competing risks of bleeding and thrombosis, complex deformity, stiffness, and compromised bone and soft tissues [4].

Most published TKA series in hemophilia lack a contemporaneous osteoarthritis (OA) comparison. Direct comparison is informative but difficult because patients with HAr are substantially younger, have different preoperative deformity, and more frequently require constrained implants. Consequently, crude comparisons of postoperative scores or complication rates may reflect differences in baseline characteristics and procedural complexity rather than the independent association of the underlying diagnosis with outcome [5–7].

In this retrospective cohort study, we compared the clinical effectiveness and perioperative safety of primary TKA performed for HAr with those of TKA performed for OA. The principal functional outcome was the Knee Society clinical score at 12 months, evaluated with adjustment for its preoperative value and other relevant covariates. The primary safety outcome was receipt of perioperative allogeneic red blood cell transfusion, considered a clinically relevant measure of perioperative bleeding burden. We also calculated perioperative blood loss and compared postoperative complications and 90-day unplanned readmissions using absolute risks, risk differences, and corresponding confidence intervals. We hypothesized that TKA would provide substantial functional improvement in patients with HAr, but that this improvement would be accompanied by greater perioperative resource use and selected complication risks than in patients with OA.

## Materials and methods

### Patients and study design

**Fig. 1:**
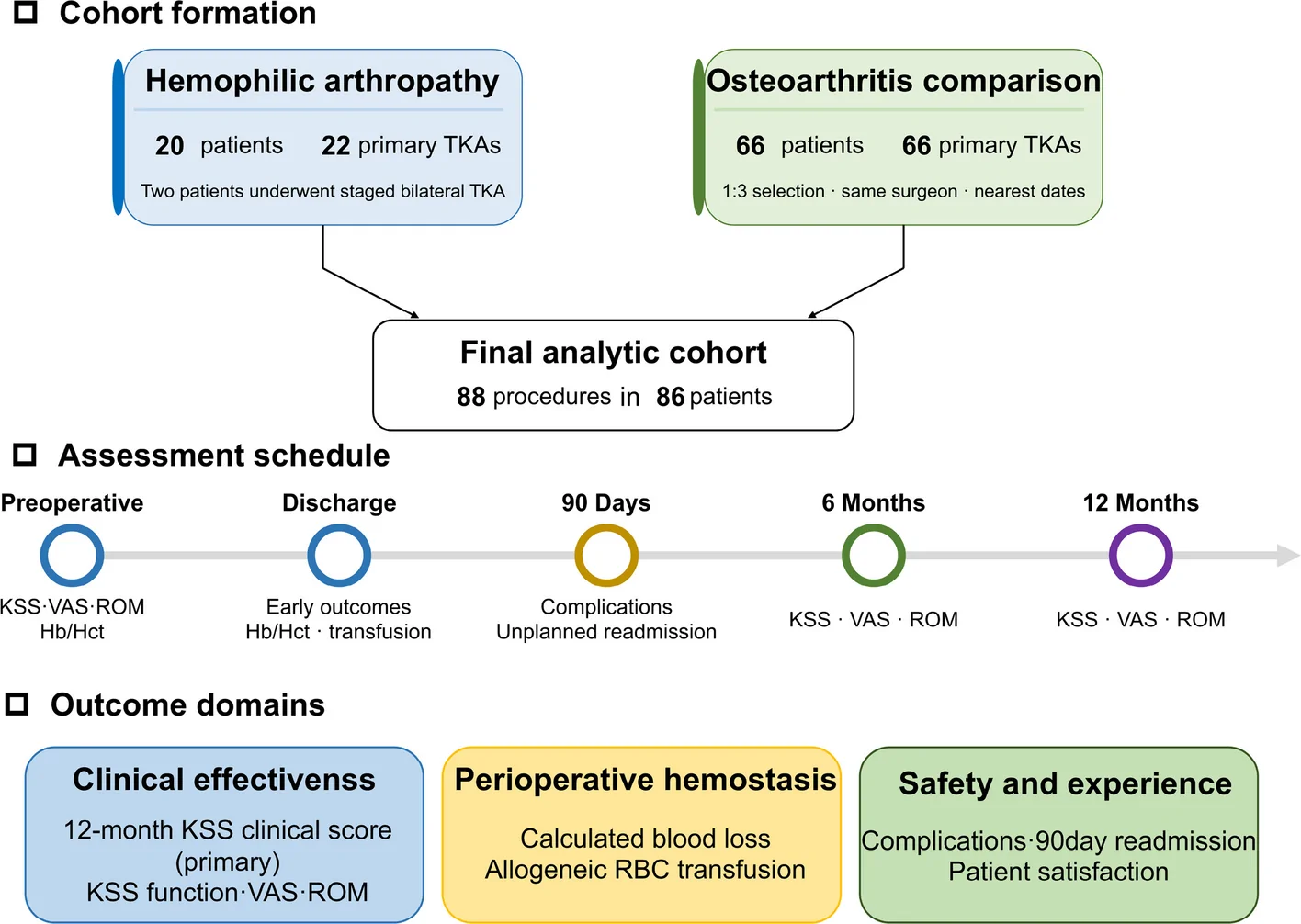
Flowchart of study design

This single-center retrospective cohort was reported in accordance with STROBE [8]. The retrospective cohort comprised primary TKAs performed from September 2021 through May 2023: 22 procedures in 20 patients with HAr and 66 procedures in 66 OA controls. Two patients with hemophilia underwent staged bilateral procedures. All participants were male, and all operations were performed by the same senior surgeon (Fig. 1).

Inclusion criteria for the HAr cohort were: (I) end-stage knee HAr and loss of mobility; (II) only TKA surgery performed during hospitalization. Exclusion criteria included: (I) additional surgery performed concurrently with TKA; (II) failure to implement comprehensive blood management measures; (III) incomplete clinical data. Inclusion criteria for the OA cohort were: (I) TKA performed for end-stage OA; (II) similar surgery day to an HAr patient; (III) the same surgeon as in the HAr cohort. Exclusion criteria included: (I) lost to follow-up; (II) incomplete clinical data. The demographic data for all included patients are presented in Table 1. The controls were selected by frequency selection rather than individual matching. Age and baseline outcome balance were not achieved and were addressed analytically. Arnold-Hilgartner stage was retained only for descriptive review within HAr and was not applied to OA.

**Table 1:** Demographic and clinical characteristics of patients

**Characteristic**	**HAr**	**OA**	**HAr-OA difference (95% CI)**	***p* value**
Patients, n	20	66	-	-
TKA procedures, n	22	66	-	-
Male sex, n/N (%)	20/20 (100%)	66/66 (100%)	-	-
Age, years	41.86 ± 11.29	68.33 ± 6.70	− 26.47 (− 31.70 to − 21.24)	< 0.001
BMI, kg/m^2^	23.70 ± 4.25	25.48 ± 3.28	− 1.78 (− 3.81 to 0.25)	0.083
Follow-up, months	21.91 ± 6.21	23.32 ± 6.14	− 1.41 (− 4.50 to 1.68)	0.361
Right knee, n/N (%)	14/22 (63.6%)	29/66 (43.9%)	19.7% (− 4.2% to 39.8%)	0.142
Higher-constraint or stemmed construct, n/N (%)	10/22 (45.5%)	1/66 (1.5%)	43.9% (24.3% to 63.9%)	< 0.001
Hemophilia A/B procedures, n	13/9	Not applicable	-	-

Values are mean ± SD unless otherwise stated. Continuous variables were compared using Welch’s t-test and categorical variables using Fisher’s exact test. Differences are HAr minus OA

*BMI* body mass index, *CI* confidence interval, *HAr* hemophilic arthropathy, *OA* osteoarthritis, *TKA* total knee arthroplasty

No prospective sample-size calculation was performed. All eligible HAr procedures were included, and three controls were selected per procedure to improve precision. Analyses of rare complications were explicitly exploratory.

The study received approval from the Institutional Review Board (Approval Number: 2024-RE-136).

### Perioperative hemostatic and general management

Hemophilia severity was defined from the lowest documented endogenous pre-treatment factor activity as severe (< 1 IU/dL), moderate (1–5 IU/dL), or mild (> 5 to < 40 IU/dL). Because untreated baseline FVIII:C or FIX:C was not consistently retained in the analytical dataset and perioperative measurements could be influenced by recent factor administration, a numerical severity distribution was not reconstructed from perioperative factor levels. The 20-patient cohort comprised 11 patients with hemophilia A and 9 with hemophilia B. All patients underwent preoperative Bethesda inhibitor testing, and none was classified as inhibitor-positive by the treating laboratory.

Perioperative hemostasis was jointly managed by orthopedic and hematology teams using a WFH-informed institutional protocol [1, 9]. Patients with hemophilia A received recombinant factor VIII, whereas those with hemophilia B received high-purity factor IX concentrate. A weight-based loading dose was individualized according to the pre-infusion factor activity, the target activity, and expected incremental recovery. Target FVIII:C or FIX:C activity was 80–100 IU/dL before incision and intraoperatively, 60–80 IU/dL on postoperative days 1–3, 40–60 IU/dL on days 4–6, and 30–50 IU/dL on days 7–14 (Supplementary Table S1). After loading, factor was administered by intermittent intravenous bolus every 12 h for factor VIII and every 24 h for factor IX. Peak activity was measured 30 min after the initial infusion; postoperative trough activity was measured at least every 3 days, and subsequent doses were adjusted to the measured level. Doses were reduced stepwise according to factor activity and wound healing. Scheduled perioperative factor replacement was discontinued after suture removal.

Prophylactic antibiotics were administered 0.5 h before surgery. Tranexamic acid was administered intravenously at a dose of 1 g half an hour before skin incision, and an additional 2 g was injected intra-articularly before wound closure. The drainage tube was usually removed on the second postoperative day. The postoperative transfusion threshold was set at 80 g/L. However, if anemia-related symptoms such as dizziness, fatigue, or palpitations occurred, the threshold could be raised to 100 g/L. Patients with OA received mechanical prophylaxis and enoxaparin 40 mg once daily, whereas patients with HAr received mechanical prophylaxis (graduated compression stockings) without routine pharmacologic thromboprophylaxis. Both cohorts underwent routine perioperative duplex ultrasonography during the index admission (within 3 days before surgery and on postoperative day 1), whereas the HAr pathway also included additional 2-week postoperative ultrasonography and D-dimer testing, irrespective of symptoms.

### Surgical technique and implant selection

**Fig. 2:**
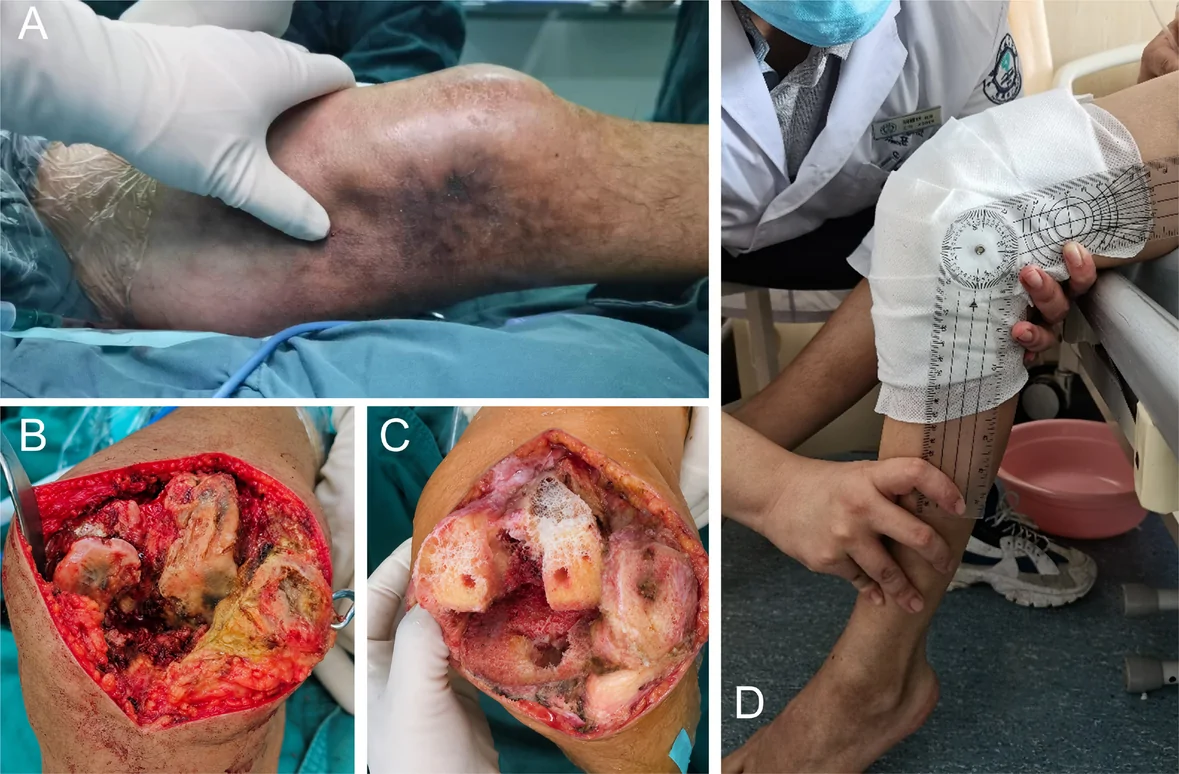
**A** Preoperative physical examination showed that the hemophilia patient’s knee joint was in a state of flexion contracture. **B**–**C** Intraoperative images of the knee before (**B**) and after (**C**) osteotomy. **D** Postoperative physical examination showed a significant improvement in the patient’s knee joint function

General anesthesia was administered to all patients. All surgeries were performed by the same senior specialist. Patients were positioned supine on the operating table (Fig. 2A). We performed the procedures in accordance with the standard protocols for TKA surgery. A tourniquet was routinely used in all TKA procedures. The tourniquet was applied at the root of the thigh before the incision and deflated after wound closure to ensure hemostasis. Following proper disinfection, the skin, subcutaneous tissue, and deep fascia were carefully dissected layer by layer through a midline anterior knee incision. The joint capsule was incised along the medial arc of the patella, with the incision kept 0.5 cm from the medial edge of the patella. All patients underwent peripheral denervation of the patella, and the articular surface of the patella was trimmed. The hemosiderin-deposited synovium, residual cruciate ligaments, menisci, and osteophytes around the articular surface were removed, especially in patients with HAr (Fig. 2B, C). Extramedullary tibial localization and proximal tibial osteotomy were performed first. Osteophytes from the posterior femoral condyle were removed, and the posterior capsule was released when necessary. The flexion and extension gaps were assessed during the procedure. Care was taken to avoid any injury to the posterior vessels and nerves. A conventional fixed-platform prosthesis was used when intraoperative testing confirmed joint stability. A semi-restricted or restricted prosthesis was only considered if the knee joint was clearly unstable. A total of 12 knees received posterior-stabilized (PS) prostheses (Zimmer Gemini), while 10 knees were implanted with constrained condylar knee (CCK) prostheses (Zimmer CCK). Because constraint may reflect baseline deformity as well as affect recovery, it was added in a sensitivity model rather than interpreted as a randomized treatment.

The implant selection by cohort is shown in Supplementary Table S2.

### Postoperative rehabilitation activities

A compression bandage and intermittent ice packs were applied to limit intra-articular bleeding and reduce pain. Postoperative rehabilitation was initiated within 24 h of surgery after confirmation of medical stability. During hospitalization, both cohorts received the same standardized rehabilitation program under the supervision of the institutional rehabilitation team (Fig. 2D). The program was delivered twice daily, 5 days per week, with each session lasting approximately 1 h, and included ankle-pump exercises, quadriceps isometric contractions, active-assisted and active knee flexion and extension, transfer training, progressive ambulation with a walker or crutches, and subsequent gait and stair training. Weight bearing as tolerated was permitted from postoperative day 1 unless otherwise restricted by the operating surgeon. Exercise intensity and progression were individualized according to pain, swelling, wound condition, quadriceps control, and functional tolerance. In the HAr cohort, rehabilitation was additionally coordinated with coagulation-factor replacement, and exercise intensity was modified or temporarily withheld if bleeding, increasing swelling, or wound concerns occurred. After discharge, outpatient therapy was prescribed three times weekly for at least 12 weeks, with an expected total rehabilitation duration of 3–5 months for HAr patients.

To monitor adherence, all patients were added to a dedicated WeChat group after discharge. Through this platform, they were asked to upload videos of their home exercises regularly. A physiotherapist reviewed the videos and provided individualized feedback. The same rehabilitation protocol and adherence monitoring system were applied to both cohorts. An additional protocol-specific difference was that patients in the HAr group received factor replacement before each physiotherapy session to maintain adequate factor levels (≥ 40%).

### Perioperative hemorrhage evaluation

Preoperative and postoperative hemoglobin (Hb) and hematocrit (Hct) values, together with perioperative allogeneic red blood cell transfusion, were collected for each TKA procedure. Calculated blood loss was estimated using the Nadler-Gross method. Because all participants were male, estimated blood volume was calculated using the male-specific Nadler equation [10]: Estimated blood volume (L) = 0.3669 × height (m)^3^ + 0.03219 × weight (kg) + 0.6041. Calculated blood loss was subsequently determined using the Gross formula [11]: Calculated blood loss (mL) = estimated blood volume (L) × 1000 × (preoperative Hct − postoperative Hct)/mean Hct, where mean Hct was the arithmetic mean of the preoperative and postoperative values. The preoperative Hct was the last available measurement before surgery, and the postoperative Hct was obtained on postoperative day 3. As a sensitivity analysis, blood loss was additionally estimated by assuming a circulating blood volume of 70 mL/kg: Calculated blood loss (mL) = 70 × body weight (kg) × (preoperative Hct − postoperative Hct)/preoperative Hct.

Receipt of perioperative allogeneic red blood cell transfusion was analyzed as a separate binary outcome. Transfused blood volume was not added to either calculated blood-loss estimate.

### Outcome measurement

**Fig. 3:**
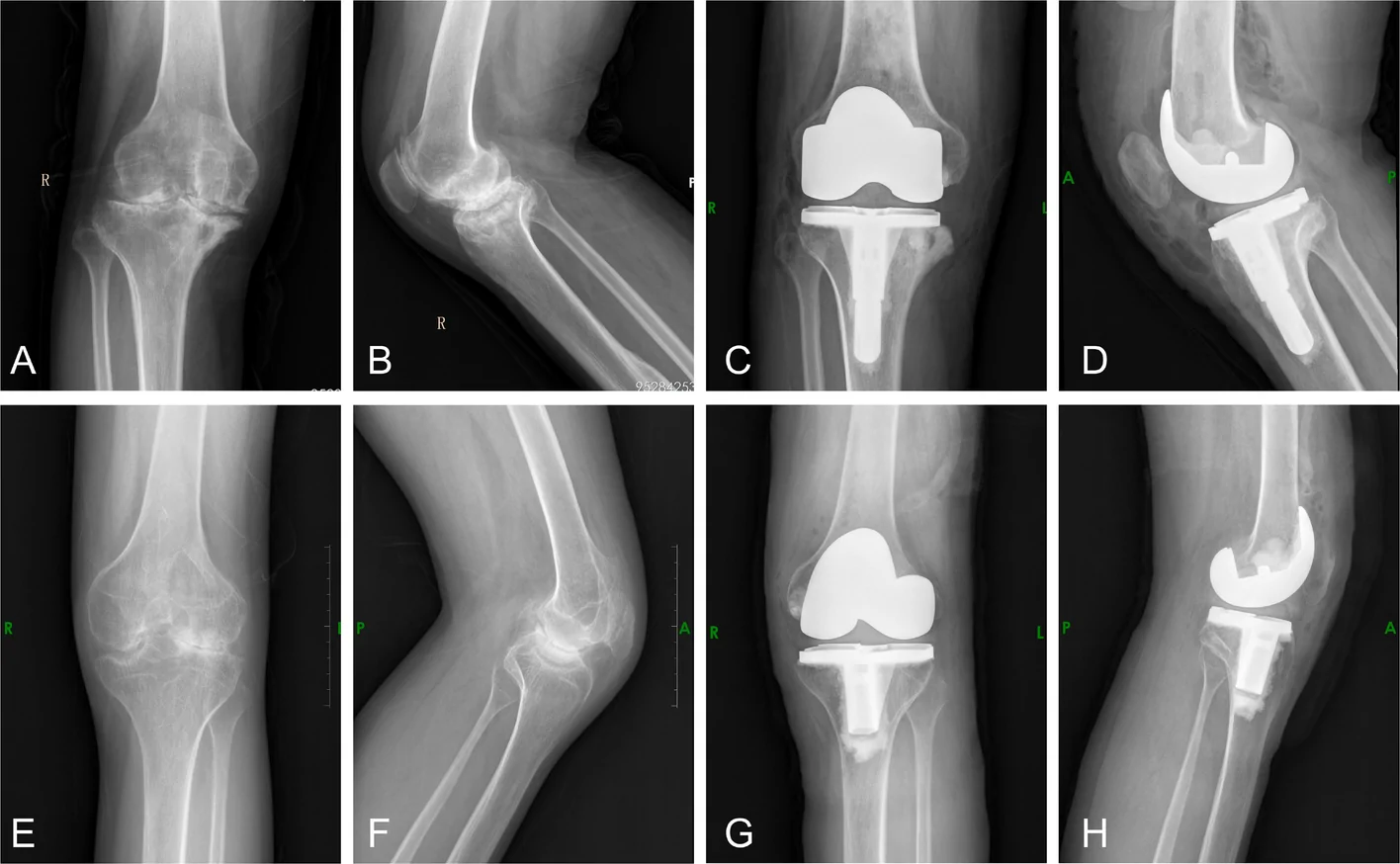
**A**–**D** Preoperative and postoperative anteroposterior and lateral X-rays of the knee joint in a patient with hemophilia A. **E**–**H** Preoperative and postoperative anteroposterior and lateral X-rays of the knee joint in a patient with hemophilia B

The Knee Society clinical and function scores, visual analogue scale (VAS) pain scores at rest and during movement, and knee range of motion (ROM), including maximum flexion and extension, were assessed preoperatively, at hospital discharge, and at 6 and 12 months after TKA. The Knee Society clinical score at 12 months was the primary functional outcome; the remaining measures and assessment time points were considered secondary outcomes. Follow-up beyond 12 months was used for surveillance of complications and subsequent procedures (Fig. 3). The mean duration of follow-up was reported descriptively and was not interpreted as a standardized 24-month outcome assessment.

### Postoperative complications and unplanned readmission

Postoperative complications were retrospectively ascertained from inpatient progress notes, nursing wound records, outpatient and emergency department records, microbiology and imaging reports, and readmission records. Potential events were independently reviewed by two investigators according to predefined operational criteria, with disagreements resolved by consensus (Supplementary Tables S3 and S4).

Poor incision healing was defined as incomplete epithelialization, persistent wound drainage beyond postoperative day 3, wound-edge separation or dehiscence, or skin-edge necrosis occurring within 30 days after TKA and requiring additional wound care, prolonged dressing changes, resuturing, or delayed suture removal, without meeting the criteria for a surgical-site infection. Transient bruising, isolated swelling, and uncomplicated delayed suture removal were not counted.

A clinically significant postoperative hematoma was defined as an operative-site or intra-articular blood collection occurring within 30 days after TKA that was documented by the treating surgeon and was supported by physical examination, ultrasonography, aspiration, or operative findings, and that required additional observation, factor replacement, aspiration, interruption of rehabilitation, or surgical treatment. Minor ecchymosis and an isolated decrease in Hb without a documented blood collection were not classified as hematoma.

Incision-related infection was defined as a superficial incisional surgical-site infection occurring within 30 days after TKA and involving only the skin or subcutaneous tissue of the incision. In accordance with the Centers for Disease Control and Prevention/National Healthcare Safety Network criteria [12], at least one of the following was required: purulent drainage from the superficial incision; identification of an organism from an aseptically obtained superficial-incision specimen; deliberate opening, re-access, or aspiration of the incision accompanied by local pain or tenderness, swelling, erythema, or heat and treatment with antibiotics for at least 2 calendar days; or an explicit diagnosis of superficial incisional surgical-site infection by the treating physician. Cellulitis alone, a stitch abscess alone, and aseptic wound-healing problems were not classified as infection. Superficial incisional infection was analyzed separately from periprosthetic joint infection.

Calf muscular-vein thrombosis was assessed from postoperative lower-extremity duplex ultrasound reports obtained during the index admission or within 90 days after surgery. It was defined as thrombus confined to a gastrocnemius or soleal vein, demonstrated by partial or complete non-compressibility, visible intraluminal thrombus or a color-Doppler filling defect, with or without reduced venous flow. Muscular-vein thrombosis was reported separately from proximal deep-vein thrombosis and pulmonary embolism.

An unplanned 90-day readmission was defined as any non-elective inpatient admission occurring after discharge from the index TKA admission and within 90 days of the index operation. Planned staged procedures, scheduled factor-replacement admissions, routine follow-up visits, and emergency-department visits not resulting in admission were excluded.

### Statistical analysis

Continuous variables were assessed for distributional characteristics using histograms, quantile–quantile plots, and Shapiro–Wilk tests and were summarized as mean (SD) or median (interquartile range), as appropriate. Categorical variables are presented as number (percentage). Unadjusted between-group comparisons of continuous outcomes were performed using Welch’s t-test, with the Mann–Whitney U test as a sensitivity analysis. Preoperative-to-12-month changes within each group were assessed using paired t-tests, with Wilcoxon signed-rank tests as sensitivity analyses. Continuous between-group effects are reported as mean differences with 95% confidence intervals (CIs).

Binary outcomes were compared using Fisher’s exact test and are reported as risks, absolute risk differences, and risk ratios with 95% CIs. CIs for risk differences were calculated using the Newcombe-Wilson method. When either group contained no events, risk ratios were estimated using the Haldane-Anscombe correction.

For this analysis, the 12-month Knee Society clinical score and receipt of perioperative allogeneic red blood cell transfusion were designated as the primary functional and safety outcomes, respectively. All other outcomes were secondary or exploratory. Twelve-month continuous outcomes were analyzed using analysis of covariance, with study group, age, body mass index, and the corresponding preoperative value included as covariates. Patient-clustered robust standard errors accounted for the inclusion of staged bilateral procedures from the same patient. Adjusted group effects are reported as mean differences with 95% CIs. Sensitivity analyses additionally adjusted for the use of a higher-constraint or stemmed implant construct and repeated the models after retaining only the first procedure for each patient in the HAr group.

Calculated blood loss was analyzed using multivariable linear regression adjusted for study group, age, body mass index, and preoperative Hct, with patient-clustered robust standard errors. Perioperative allogeneic red blood cell transfusion, wound-healing complications, 90-day unplanned readmission, and adjudicated postoperative calf muscular-vein thrombosis were examined in exploratory Firth-penalized logistic regression models including study group, age, and body mass index. Adjusted associations are reported as odds ratios with 95% CIs.

Analyses were performed using complete cases for each outcome, without imputation of missing clinical data. All tests were two-sided, and *p* < 0.05 was considered statistically significant. No adjustment was made for multiple comparisons; secondary and sensitivity analyses were therefore interpreted as exploratory. Analyses were conducted using Python version 3.12.

### Use of artificial intelligence-assisted technologies

OpenAI ChatGPT was used to assist with English-language editing and drafting analysis code. The authors independently reviewed the source data, code, statistical outputs, clinical interpretation, and final text and take full responsibility for the content of the manuscript.

## Results

### Demographic and clinical characteristics

The analysis included 22 HAr TKAs in 20 patients and 66 OA TKAs in 66 patients (Fig. 1). All participants were male. The HAr group included 13 type A and 9 type B procedures and was 26.47 years younger on average. Body mass index (BMI) and follow-up were not statistically different with Welch’s t-test. Higher-constraint or stemmed constructs were used in 45.5% versus 1.5% of procedures (Table 1).

### Perioperative hemorrhage comparison

Preoperative Hb concentrations were similar between the groups. Using the Nadler-Gross method, mean calculated blood loss was 532.53 ± 355.18 mL in the HAr group and 628.00 ± 328.38 mL in the OA group (mean difference, − 95.47 mL; 95% CI − 270.21 to 79.27; *p* = 0.275) (Table 2). After adjustment for age, BMI, and preoperative hematocrit, HAr was not independently associated with calculated blood loss (adjusted mean difference versus OA, 148.80 mL; 95% CI − 125.77 to 423.38; *p* = 0.284) (Supplementary Table S5). The alternative 70-mL/kg calculation yielded consistent findings (507.33 ± 332.78 versus 626.71 ± 321.81 mL; mean difference, − 119.38 mL; 95% CI − 284.60 to 45.85; *p* = 0.151) (Table 2).

**Table 2:** Perioperative hematological outcomes

**Outcome**	**HAr**	**OA**	**HAr-OA difference (95% CI)**	***p* value**
Preoperative Hb, g/L	140.05 ± 15.98	134.18 ± 14.53	5.86 (− 1.96 to 13.69)	0.137
Postoperative Hb, g/L	126.91 ± 16.98	119.08 ± 15.25	7.83 (− 0.47 to 16.14)	0.064
Calculated blood loss, Nadler-Gross method, mL	532.53 ± 355.18	628.00 ± 328.38	− 95.47 (− 270.21 to 79.27)	0.275
Calculated blood loss, 70-mL/kg method, mL	507.33 ± 332.78	626.71 ± 321.81	− 119.38 (− 284.60 to 45.85)	0.151
Perioperative allogeneic red blood cell transfusion, n/N (%)	4/22 (18.2%)	0/66 (0.0%)	18.2% (6.0% to 38.5%)	0.003

Values are mean ± SD or n/N (%). The Nadler-Gross estimate was the primary calculated blood-loss measure; the 70-mL/kg estimate was a sensitivity analysis. *p*-values are from Welch’s t-test for continuous outcomes and Fisher’s exact test for transfusion

*CI* confidence interval, *HAr* hemophilic arthropathy, *OA* osteoarthritis

Perioperative allogeneic red blood cell transfusion was administered in four HAr procedures and no OA procedures (*p* = 0.003) (Table 2). In the exploratory Firth logistic regression model adjusted for age and body mass index, the estimated association was highly imprecise because of the small number of transfusion events (adjusted odds ratio, 20.73; 95% CI 0.47–913.62; *p* = 0.116) (Supplementary Table S6).

### Clinical outcomes improvement

Knee Society clinical and function scores improved significantly from baseline to 12 months in both groups. The mean increase in the Knee Society clinical score was 49.64 ± 18.43 points in the HAr group and 53.24 ± 5.77 points in the OA group (Table 3). The unadjusted between-group change-score difference was − 3.61 points (95% CI − 11.88 to 4.67; *p* = 0.376). After adjustment for age, body mass index and the corresponding preoperative score, the 12-month outcomes did not differ significantly between HAr and OA for either the Knee Society clinical score (adjusted mean difference, 0.54 points; 95% CI − 4.19 to 5.28; *p* = 0.820) or the Knee Society function score (adjusted mean difference, 3.02 points; 95% CI − 9.73 to 15.77; *p* = 0.639) (Table 3).

**Table 3:** Comparison of clinical outcomes from baseline to 12 months in two groups

**Outcome**	**HAr preoperative**	**HAr 12 months**	**HAr change**	**OA preoperative**	**OA 12 months**	**OA change**	**Unadjusted change-score difference (95% CI)**	**Adjusted HAr-OA mean difference at 12 months (95% CI)**	**Adj. *p* value**
KSS clinical score	38.86 ± 16.74	88.50 ± 7.58	49.64 ± 18.43	38.02 ± 5.25	91.26 ± 2.30	53.24 ± 5.77	− 3.61 (− 11.88 to 4.67)	0.54 (− 4.19 to 5.28)	0.820
KSS function score	16.82 ± 22.23	75.91 ± 21.36	59.09 ± 28.85	19.45 ± 14.46	88.17 ± 8.00	68.71 ± 12.87	− 9.62 (− 22.74 to 3.49)	3.02 (− 9.73 to 15.77)	0.639
VAS pain at rest	0.14 ± 0.64	0.00 ± 0.00	− 0.14 ± 0.64	2.64 ± 2.75	0.00 ± 0.00	− 2.64 ± 2.75	2.50 (1.77 to 3.23)	-	-
VAS pain during movement	6.41 ± 2.22	0.00 ± 0.00	− 6.41 ± 2.22	8.32 ± 1.34	0.23 ± 0.55	− 8.09 ± 1.60	1.68 (0.63 to 2.73)	− 0.54 (− 0.92 to − 0.17)	0.005
Knee flexion, degrees	54.77 ± 40.69	80.91 ± 32.35	26.14 ± 36.84	78.29 ± 11.78	116.62 ± 5.73	38.33 ± 12.91	− 12.20 (− 28.78 to 4.39)	− 19.64 (− 38.29 to − 0.99)	0.039
Knee extension, degrees	− 7.62 ± 13.00	− 2.95 ± 5.91	5.00 ± 11.83	− 2.35 ± 6.86	− 0.30 ± 1.73	2.05 ± 6.50	2.95 (− 2.63 to 8.53)	0.66 (− 2.34 to 3.65)	0.663

Values are mean ± SD. Adjusted mean differences were estimated using analysis of covariance with study group, age, BMI, and the corresponding preoperative value, with patient-clustered robust standard errors. Resting VAS at 12 months was zero in both groups, and the adjusted effect was therefore not estimable

*BMI* body mass index, *CI* confidence interval, *HAr* hemophilic arthropathy, *KSS* Knee Society Score, *OA* osteoarthritis, *VAS* visual analogue scale

**Fig. 4:**
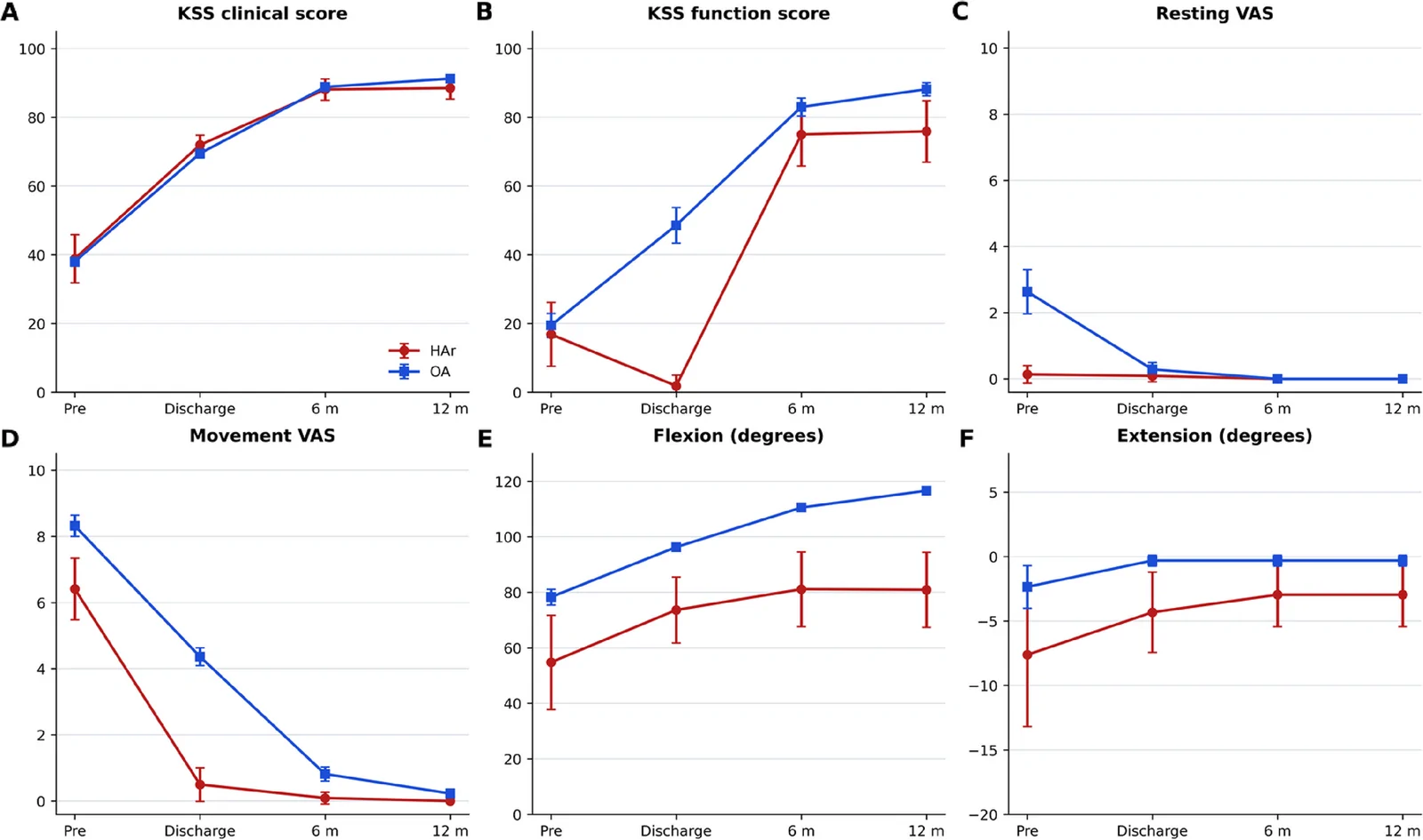
Trends in the KSS clinical and function score (**A**–**B**), VAS rest and motion score (**C**–**D**), flexion and extension ROM (**E**–**F**) with the results obtained through 12 months postoperatively. Points are means and error bars are 95% confidence intervals. HAr, hemophilic arthropathy; OA, osteoarthritis

At 12 months, the HAr group had lower adjusted knee flexion than the OA group (adjusted mean difference, − 19.64°; 95% CI − 38.29° to − 0.99°; *p* = 0.039) (Table 3). Knee extension did not differ significantly between the groups. Pain during movement was lower in the HAr group (adjusted mean difference in VAS score, − 0.54; 95% CI − 0.92 to − 0.17; *p* = 0.005) (Table 3). Additional adjustment for the use of a higher-constraint or stemmed implant construct yielded consistent findings (Table 3, Supplementary Table S5 and Fig. 4).

### Complications and satisfaction

Wound-healing complications occurred after 6 of 22 procedures (27.3%) in the HAr group and 3 of 66 procedures (4.5%) in the OA group, corresponding to an absolute risk difference of 22.7% (95% CI 6.5% to 43.8%; *p* = 0.007). Unplanned readmission within 90 days occurred after 9 of 22 procedures (40.9%) and 1 of 66 procedures (1.5%), respectively (risk difference, 39.4%; 95% CI 20.6% to 59.8%; *p* < 0.001) (Table 4). In exploratory Firth logistic regression models adjusted for age and body mass index, the associations remained highly imprecise for both wound-healing complications (adjusted odds ratio, 2.63; 95% CI 0.21–33.16; *p* = 0.455) and 90-day readmission (adjusted odds ratio, 2.92; 95% CI 0.16–53.96; *p* = 0.472) (Supplementary Table S6).

**Table 4:** Complications and satisfaction in two cohorts

**Outcome**	**HAr, n/N (%)**	**OA, n/N (%)**	**Risk difference (95% CI)**	***p* value**
Incision-related infection	0/22 (0.0%)	2/66 (3.0%)	− 3.0% (− 10.4% to 12.0%)	1.000
Wound-healing complication	6/22 (27.3%)	3/66 (4.5%)	22.7% (6.5% to 43.8%)	0.007
Hematoma	2/22 (9.1%)	0/66 (0.0%)	9.1% (0.5% to 27.8%)	0.060
Persistent neurological deficit at 12 months	0/22 (0.0%)	3/66 (4.5%)	− 4.5% (− 12.5% to 10.6%)	0.570
Postoperative calf muscular-vein thrombosis	2/22 (9.1%)	6/66 (9.1%)	0.0% (− 11.4% to 19.3%)	1.000
Periprosthetic joint infection	0/22 (0.0%)	0/66 (0.0%)	0.0% (− 5.5% to 14.9%)	1.000
Unplanned readmission within 90 days	9/22 (40.9%)	1/66 (1.5%)	39.4% (20.6% to 59.8%)	< 0.001
Reoperation	1/22 (4.5%)	3/66 (4.5%)	0.0% (− 8.8% to 17.5%)	1.000
Satisfied or very satisfied	20/22 (90.9%)	64/66 (97.0%)	− 6.1% (− 24.9% to 3.8%)	0.259

*p* values are from Fisher’s exact test. Risk differences are HAr minus OA

*CI* confidence interval, *HAr* hemophilic arthropathy, *OA* osteoarthritis

Postoperative calf muscular-vein thrombosis was recorded after 2 of 22 HAr procedures and 6 of 66 osteoarthritis procedures (*p* = 1.000) (Table 4). Two early postoperative superficial incisional infections were recorded in the OA group. However, no periprosthetic joint infection was identified in either group. Satisfaction or high satisfaction was reported after 20 of 22 HAr procedures (90.9%) and 64 of 66 osteoarthritis procedures (97.0%) (Table 4).

## Discussion

In this retrospective comparative cohort study, primary TKA was associated with substantial improvements in Knee Society clinical and function scores among patients with HAr. After adjustment for baseline outcome values, age, and body mass index, the 12-month Knee Society scores did not differ significantly from those of male patients undergoing TKA for OA. Nevertheless, patients with HAr had lower postoperative knee flexion, indicating that meaningful improvement in global knee function may coexist with residual limitations in ROM. Calculated blood loss was not significantly greater in HAr, although perioperative allogeneic red blood cell transfusion was more frequent. Crude rates of wound-healing complications and 90-day readmission were also higher, but the adjusted estimates were highly imprecise because of the small number of events and the limited overlap in baseline characteristics.

The marked age difference between the groups is core to the interpretation of these findings. Patients with HAr underwent TKA more than two decades earlier than patients with OA, reflecting the distinct natural histories of the two conditions. Although covariate adjustment reduced measured confounding, regression modelling cannot fully compensate for limited age overlap between groups and may require extrapolation beyond the observed covariate distributions. Selection of OA comparators according to sex, surgical period and surgeon improved comparability for these characteristics but did not constitute individual matching. The adjusted estimates should therefore be interpreted as associations rather than causal effects of the underlying diagnosis.

Both groups achieved large improvements in Knee Society scores, and no clear adjusted difference in the 12-month clinical or function scores was detected. However, postoperative flexion remained lower in the HAr group. This finding is consistent with the greater prevalence of longstanding contracture, fixed deformity, soft-tissue fibrosis, and complex reconstruction described in previous hemophilia series [4, 13]. Lower movement-pain scores were also observed in patients with HAr. Because pain reporting may be influenced by preoperative disease experience, activity level, and postoperative ROM, this difference should be interpreted as an observed outcome rather than evidence of greater pain tolerance.

The substantial imbalance in implant selection further reflects differences in disease severity and operative complexity. Higher-constraint and stemmed constructs were used more frequently in HAr and may influence postoperative recovery and ROM. However, implant constraint is selected in response to deformity, instability, and bone loss and may therefore function as both a marker of preoperative severity and an intermediate factor in the relationship between diagnosis and outcome. Additional adjustment for implant construct did not materially alter the Knee Society score estimates and strengthened the negative association with postoperative flexion. These sensitivity analyses should not be interpreted as causal comparisons between implant designs.

Calculated blood loss did not differ significantly between the groups using either the Nadler-Gross method or the alternative weight-based calculation. In contrast, allogeneic red blood cell transfusion occurred only in the HAr group. Calculated blood loss and transfusion measure related but distinct aspects of perioperative hemostasis: Hct-based estimates are influenced by fluid administration, laboratory timing, and transfusion, whereas the decision to transfuse may also reflect symptoms, hemodynamic status, institutional thresholds and clinical concern regarding continued bleeding. The present findings therefore support the conclusion that calculated blood loss was not increased but that transfusion requirements were greater in patients with HAr. This distinction reinforces the importance of individualized hemostatic planning, monitored factor replacement and close multidisciplinary perioperative management [9, 14–16].

The higher crude frequencies of wound-healing complications and unplanned readmission warrant clinical attention. However, the adjusted estimates had extremely wide confidence intervals and did not establish that HAr was independently associated with either outcome. These events may reflect a combination of bleeding-related concerns, wound surveillance, factor-replacement requirements, comorbidity, operative complexity, and different thresholds for hospital reassessment. Larger multicenter cohorts with prospectively defined complications are required to determine whether these signals represent reproducible differences in perioperative risk.

Distal thromboses detected by routine screening may differ clinically from symptomatic proximal deep-vein thrombosis or pulmonary embolism. Thrombotic outcomes should therefore be interpreted according to anatomical location, symptoms, screening strategy, and timing. Current guidance supports individualized assessment of thrombotic and bleeding risks in patients with hemophilia rather than routine extrapolation from conventional arthroplasty pathways [1, 17–19].

Despite differences in perioperative events and postoperative flexion, patient-reported satisfaction was high in both groups. This finding, together with the substantial improvements in Knee Society scores, suggests that TKA can provide meaningful clinical benefit for appropriately selected patients with end-stage HAr when performed within a multidisciplinary care pathway [13].

This study has several limitations. First, its retrospective, single-center design is susceptible to selection bias, measurement error, and residual confounding. Second, the HAr group comprised only 22 procedures in 20 patients, limiting statistical power, particularly for uncommon complications and multivariable binary-outcome models. Third, the pronounced differences in age and implant construct resulted in limited covariate overlap between the groups and could not be fully addressed through statistical adjustment. Fourth, complete-case and sensitivity analyses reduced but could not eliminate the potential for measurement bias. Fifth, although the institutional factor-replacement schedule and monitoring pathway were verified, untreated baseline factor activity was not consistently available for severity classification, and patient-level factor consumption and product brand were not available for standardized comparative analysis. Finally, standardized functional assessment was limited to 12 months, and subsequent follow-up was used only for complication surveillance. Longer-term implant survivorship and functional outcomes therefore require further investigation.

## Conclusions

Primary TKA was associated with substantial 12-month clinical and functional improvement in patients with HAr. After adjustment for baseline characteristics, Knee Society clinical and function scores did not differ significantly from those of male OA controls, although postoperative knee flexion remained lower. Calculated blood loss was not significantly increased, whereas allogeneic red blood cell transfusion was more frequent. The higher crude frequencies of wound-healing complications and 90-day readmission require confirmation in larger, prospectively defined multicenter studies. These findings support TKA as an effective treatment for end-stage HAr while emphasizing the importance of multidisciplinary perioperative management and cautious interpretation of comparative safety outcomes.

## Supplementary Information

Supplementary Material 1. 

## Data Availability

The datasets used and/or analysed during the current study are available from the corresponding author on reasonable request.

## Abbreviations

TKA: Total knee arthroplasty
HAr: Hemophilic arthropathy
OA: Osteoarthritis
PS: Posterior-stabilized
CCK: Constrained condylar knee
Hb: Hemoglobin
Hct: Hematocrit
BMI: Body Mass Index
VAS: Visual Analog Scale
ROM: Range of Motion
THA: Total hip arthroplasty
KSS: Knee Society Score
