# Supplementary material for: Clinical outcomes and perioperative safety of primary total knee arthroplasty for hemophilic arthropathy versus osteoarthritis: a retrospective cohort study
Source: Arthroplasty. 2026 Sep 18;8:71. doi: 10.1186/s42836-026-00431-5 (PMC13587490; doi:10.1186/s42836-026-00431-5)
Supplement: Supplementary file 1 [file 42836_2026_431_MOESM1_ESM.docx]

**Supplementary information**

**Table S1.** Perioperative coagulation factor replacement protocol

| **Hemophilia type** | **Factor product and dosing interval** | **Perioperative period** | **Target activity, IU/dL** |
| --- | --- | --- | --- |
| Hemophilia A | Recombinant factor VIII; intravenous bolus every 12 h | Preoperative and intraoperative | 80–100 |
|  |  | Postoperative days 1–3 | 60–80 |
|  |  | Postoperative days 4–6 | 40–60 |
|  |  | Postoperative days 7–14 | 30–50 |
| Hemophilia B | High-purity factor IX concentrate; intravenous bolus every 24 h | Preoperative and intraoperative | 80–100 |
|  |  | Postoperative days 1–3 | 60–80 |
|  |  | Postoperative days 4–6 | 40–60 |
|  |  | Postoperative days 7–14 | 30–50 |

*Note*: A weight-based loading dose was individualized according to pre-infusion factor activity, the target activity, and expected incremental recovery. Peak activity was measured 30 minutes after the initial infusion; trough activity was measured at least every 3 days. Subsequent doses were adjusted to the measured level and tapered according to factor activity and wound healing until discontinuation after suture removal. All patients underwent preoperative Bethesda inhibitor testing, and none was classified as inhibitor-positive by the treating laboratory. The institutional targets were informed by WFH guidance; the common factor IX pathway used targets that were not lower than the higher-dose WFH practice-pattern ranges.

**Table S2.** Implant construct categories

| **Construct category** | **HAr, n/N (%)** | **OA, n/N (%)** |
| --- | --- | --- |
| Standard primary construct | 12/22 (54.5%) | 65/66 (98.5%) |
| Higher-constraint or stemmed construct | 10/22 (45.5%) | 1/66 (1.5%) |

*Note*: Construct category was determined from the operative implant record. HAr, hemophilic arthropathy; OA, osteoarthritis.

**Table S3.** Outcome definitions and ascertainment

| **Outcome** | **Operational definition** |
| --- | --- |
| Incision-related infection | An infection involving only the skin or subcutaneous tissue within 30 days after TKA that met the prespecified CDC/NHSN-based criteria[1]. |
| Wound-healing complication | Incomplete epithelialization, persistent drainage beyond postoperative day 3, wound-edge separation or dehiscence, or skin-edge necrosis within 30 days that required additional wound care without meeting infection criteria. |
| Hematoma | An operative-site or intra-articular blood collection within 30 days, supported by examination, imaging, aspiration, or operative findings and requiring additional observation or treatment. |
| Persistent neurological deficit | A neurological deficit documented as persisting at the 12-month assessment. |
| Postoperative calf muscular-vein thrombosis | A postoperative thrombus confined to a gastrocnemius or soleal vein and demonstrated by duplex ultrasonography. |
| Periprosthetic joint infection | A clinician-recorded periprosthetic joint infection, analyzed separately from superficial incisional infection. |
| Unplanned 90-day readmission | Any non-elective inpatient admission after discharge and within 90 days of the index TKA; planned staged procedures and visits without admission were excluded. |
| Reoperation | Any subsequent operative procedure on the index knee during follow-up. |
| Satisfaction | Satisfied or very satisfied as documented at postoperative follow-up. |

*Note:* Outcomes were ascertained retrospectively from structured clinical records and verified against available medical notes, imaging reports, and readmission or reoperation records. TKA, total knee arthroplasty.

**Table S4.** Reasons for unplanned 90-day readmission

| **Cohort** | **Reason for readmission** | **Procedures, n** |
| --- | --- | --- |
| HAr | Bleeding or coagulopathy-related | 5 |
| HAr | Wound-healing concern | 2 |
| HAr | Trauma | 1 |
| HAr | Other orthopedic reason | 1 |
| OA | Hypertension | 1 |

*Note*: Reasons were classified from the diagnosis documented for the unplanned readmission. HAr, hemophilic arthropathy; OA, osteoarthritis.

**Table S5.** Adjusted continuous outcome models

| **Outcome** | **N/clusters** | **Base adjusted mean difference (95% CI)** | ***p* value** | **Construct-adjusted mean difference (95% CI)** | ***p* value** |
| --- | --- | --- | --- | --- | --- |
| KSS clinical score at 12 months | 88/86 | 0.54 (−4.19 to 5.28) | 0.820 | −1.56 (−7.34 to 4.22) | 0.592 |
| KSS function score at 12 months | 88/86 | 3.02 (−9.73 to 15.77) | 0.639 | −0.55 (−13.98 to 12.89) | 0.936 |
| VAS pain at rest at 12 months | 88/86 | Not estimable | - | Not estimable | - |
| VAS pain during movement at 12 months | 88/86 | −0.54 (−0.92 to −0.17) | 0.005 | −0.51 (−0.87 to −0.15) | 0.006 |
| Knee flexion at 12 months, degrees | 88/86 | −19.64 (−38.29 to −0.99) | 0.039 | −30.12 (−54.04 to −6.20) | 0.014 |
| Knee extension at 12 months, degrees | 87/85 | 0.66 (−2.34 to 3.65) | 0.663 | 0.98 (−1.66 to 3.61) | 0.462 |
| Calculated blood loss, Nadler-Gross method, mL | 87/85 | 148.80 (−125.77 to 423.38) | 0.284 | 165.04 (−121.25 to 451.33) | 0.255 |

*Note*: Base 12-month models included study group, age, BMI, and the corresponding preoperative outcome. The blood-loss model included study group, age, BMI, and preoperative hematocrit. Construct-adjusted models additionally included higher-constraint or stemmed implant use. Standard errors were clustered by patient. Effects are HAr minus OA. BMI, body mass index; CI, confidence interval; HAr, hemophilic arthropathy; KSS, Knee Society Score; OA, osteoarthritis; VAS, visual analog scale.

**Table S6.** Firth models for sparse binary outcomes

| **Outcome** | **N** | **Events** | **Adjusted OR** | **95% CI** | ***p* value** |
| --- | --- | --- | --- | --- | --- |
| Perioperative allogeneic red blood cell transfusion | 88 | 4 | 20.73 | 0.47 to 913.62 | 0.116 |
| Wound-healing complication | 88 | 9 | 2.63 | 0.21 to 33.16 | 0.455 |
| Unplanned readmission within 90 days | 88 | 10 | 2.92 | 0.16 to 53.96 | 0.472 |
| Postoperative calf muscular-vein thrombosis | 88 | 8 | 0.43 | 0.02 to 8.26 | 0.576 |

*Note*: Firth-penalized logistic regression models included study group, age, and BMI. Adjusted odds ratios compare HAr with OA. These models were exploratory because of sparse events and limited covariate overlap. BMI, body mass index; CI, confidence interval; HAr, hemophilic arthropathy; OA, osteoarthritis; OR, odds ratio.

**Table S7.** Patient-level sensitivity analyses

| **Outcome** | **N** | **Adjusted HAr-OA mean difference (95% CI)** | ***p* value** |
| --- | --- | --- | --- |
| KSS clinical score at 12 months | 86 | 0.22 (−4.61 to 5.04) | 0.929 |
| KSS function score at 12 months | 86 | 1.96 (−10.84 to 14.76) | 0.761 |
| VAS pain during movement at 12 months | 86 | −0.54 (−0.91 to −0.17) | 0.005 |
| Knee flexion at 12 months, degrees | 86 | −19.45 (−38.22 to −0.68) | 0.042 |
| Knee extension at 12 months, degrees | 85 | 0.52 (−2.48 to 3.52) | 0.731 |

*Note*: The sensitivity analysis retained only the first HAr procedure for each patient and all OA procedures. Models included study group, age, BMI, and the corresponding preoperative outcome. CI, confidence interval; HAr, hemophilic arthropathy; KSS, Knee Society Score; OA, osteoarthritis; VAS, visual analog scale.

**Reference**

1. Centers for Disease Control and Prevention. CDC/NHSN surveillance definitions for specific types of infections. 2023. https://stacks.cdc.gov/view/cdc/127235.
